# Motif mediated protein-protein interactions as drug targets

**DOI:** 10.1186/s12964-016-0131-4

**Published:** 2016-03-02

**Authors:** Carles Corbi-Verge, Philip M. Kim

**Affiliations:** Terrence Donnelly Centre for Cellular and Biomolecular Research, University of Toronto, Toronto, ON M5S 3E1 Canada; Department of Molecular Genetics, University of Toronto, Toronto, ON M5S 3E1 Canada; Department of Computer Science, University of Toronto, Toronto, ON M5S 3E1 Canada

**Keywords:** Protein-protein interactions, Linear motifs, Drug discovery, Small-molecule inhibitors, Peptidomimetics, Peptides as therapeutics

## Abstract

Protein-protein interactions (PPI) are involved in virtually every cellular process and thus represent an attractive target for therapeutic interventions. A significant number of protein interactions are frequently formed between globular domains and short linear peptide motifs (DMI). Targeting these DMIs has proven challenging and classical approaches to inhibiting such interactions with small molecules have had limited success. However, recent new approaches have led to the discovery of potent inhibitors, some of them, such as Obatoclax, ABT-199, AEG-40826 and SAH-p53-8 are likely to become approved drugs. These novel inhibitors belong to a wide range of different molecule classes, ranging from small molecules to peptidomimetics and biologicals. This article reviews the main reasons for limited success in targeting PPIs, discusses how successful approaches overcome these obstacles to discovery promising inhibitors for human protein double minute 2 (HDM2), B-cell lymphoma 2 (Bcl-2), X-linked inhibitor of apoptosis protein (XIAP), and provides a summary of the promising approaches currently in development that indicate the future potential of PPI inhibitors in drug discovery.

## Background

Proteins form the basic machinery of cells, and the precise interactions between them, known as Protein-Protein Interactions (PPIs), are fundamental for appropriate execution of all cellular mechanisms. At a high level, we can differentiate two types of interactions: one involving more stable interactions that establish macromolecular complexes, the other involving transient interactions, usually between proteins that mediate signalling pathways and regulatory process [1].

**Fig. 1:**
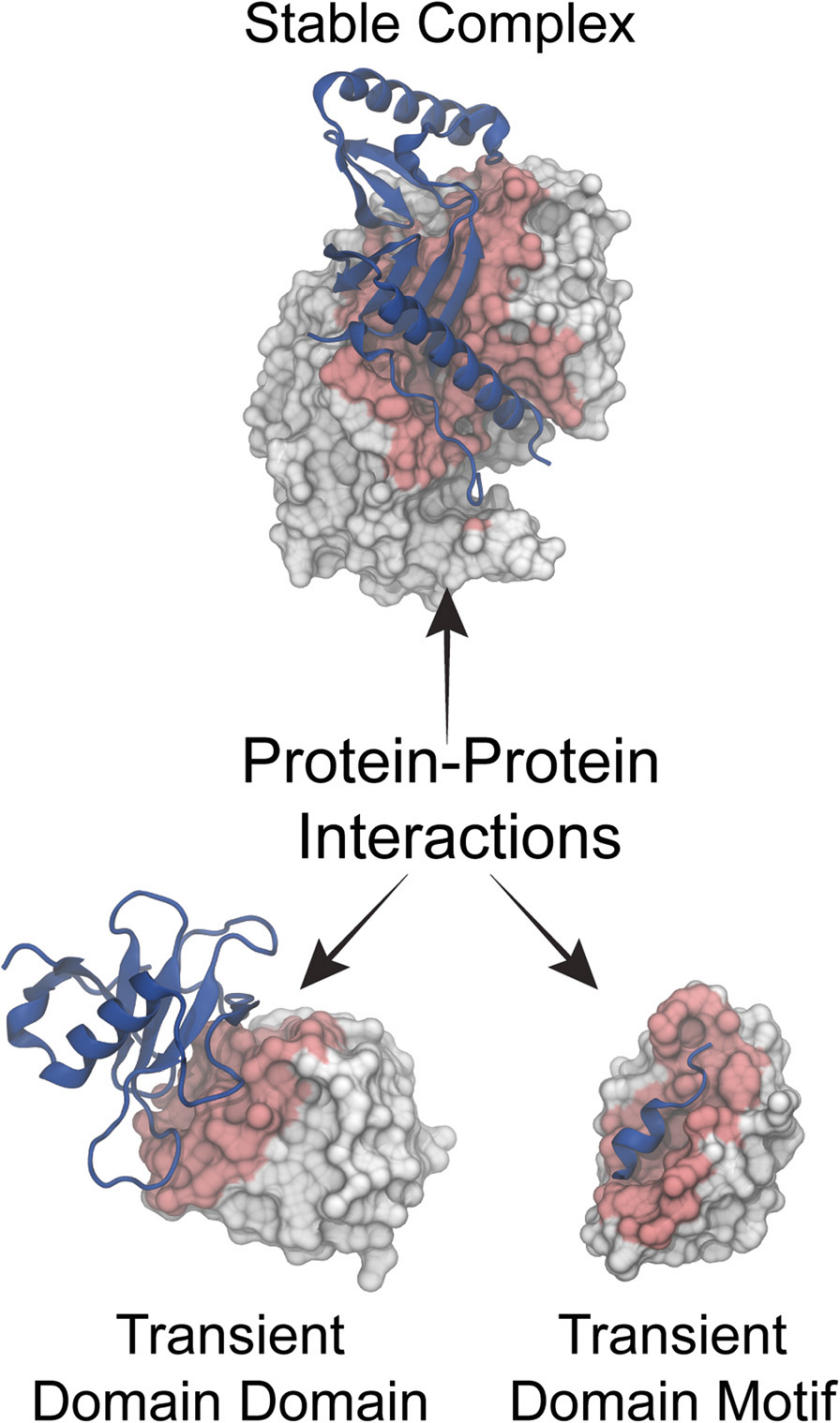
Classification of protein–protein interaction types based on affinity and stability. Stable complex (PDB: 1 F34) Structure of Ascaris pepsin inhibitor-3 bound to Porcine pepsin; Transient Domain-Domain interaction (PDB: 1AY7) Structure of the Ribonuclease SA Complex With Barstar; Transient Domain-Motif interaction (PDB: 1YCR) Structure of the MDM2 oncoprotein bound to the p53 tumour suppressor. For each complex, one of the interacting partners is displayed in blue cartoon representation, while the other is displayed in grey surface representation with the interface highlighted in red

The former group of PPI are usually mediated by reciprocal recognition interfaces at the protein surface – domain-domain interactions (DDI) – while the latter involves domain binding to a continuous binding epitope, or domain-motif interaction (DMI). Generally, a short segment or an unstructured region of the target protein contains the recognition motif [2]. These motifs can either be in terminal regions or within a loop of the target protein, and they bind to the relatively flat recognition domains using a small groove (Fig. 1).

This mechanism of recognition is very common in cellular processes, evidenced by the large number of recognition domains encoded by the human genome [1, 3–5]. There are several diseases and syndromes related to the disruption of specific DMI motifs [6–11]. For instance, Liddle’s, Noonan’s and Usher’s hereditary syndromes can be caused by mutations in the recognition motif (WW, 14-3-3 and PDZ recognition motif respectively) leading to the deregulation of important signalling pathways [12–14]. It has also been recognized that several viruses, e.g., Ebola and Rabies viruses, hijack the cell machinery using modified domain motifs interactions [15–17]. In addition, numerous oncogenic proteins either contain a motif, or recognise motif interaction sequences for which inhibition is a potential cancer treatment [11, 18]. As an illustration, over-expression of the murine double minute 2 (MDM2) protein, an E3 ubiquitin ligase, causes a decrease in the apoptotic activities of p53 through the motif FxxxWxxL [19, 20]. Other similar examples of proteins with experimentally validated and cancer related DMI include B-cell lymphoma 2 (Bcl2) [18], bacuolovirus inhibitor of apoptosis repeat (BIR) [21] and Integrin receptors [22].

DMIs have therefore been an attractive group of new drug targets, because their fine modulation would allow for numerous desirable therapeutic effects [3, 6, 23–26]. However, despite the enormous interest in targeting protein-protein interactions, developing such drugs has proven to be very challenging. The transient nature of these interactions, moderate affinity, promiscuity of recognition, and binding interface structural properties, are among the many factors that have contributed to difficulty in discovering effective inhibitors. This had led to a general sense that protein–protein interactions might not be amenable to inhibition by small molecules [3, 27–32]. A perhaps instructive counterpoint to this view is the case of protein kinases: They were also considered to be challenging to target until a few decades ago. This opinion was based on the high homology of the enzymatic site and the potent binding of the natural binder. These factors made it difficult to find molecules specific enough to exclusively inhibit the kinase involved in disease pathophysiology, with high enough affinity to compete against the ATP. Of course, currently, there are numerous kinase inhibitors on the market. Similarly, researchers have made considerable progress over recent years in finding drug molecules that disrupt protein-protein interfaces.

In this review, we describe in detail the challenges of targeting DMI interactions. Following this we review successful approaches and discuss how they overcame the challenges of targeting DMI. We present specific cases, categorized by the nature of the inhibitor (either small molecules or biologics). We do not aim to set out the detailed pros and cons of these two categories here, as there are many insightful articles that do this elsewhere [33, 34]. Finally, this review will focus on new methods for detecting and targeting DMI, promising approaches that will provide inhibitors in the future.

### The challenges of targeting domain motif interactions

Although there is little doubt that small-molecules can interfere with PPIs, there are currently only a limited number of published examples of molecules capable to inhibit DMIs. This limited success is mainly due to the following factors.

### Complex, transient and promiscuous interactions

As is mentioned above, the majority of DMIs are involved in signalling, with moderate binding affinities. This is important for precise control of the transmitted signals, but makes their capture difficult, in particular in high-throughput screens, where the majority of our data stems from. This, together with the complexity of signal pathways, makes identification of all the partners of critical proteins in a key cellular process a challenging goal. While recent methodologies have helped to provide increasing information, many such interactions remain undetected and thus can’t be inhibited for therapeutic effect [35, 36].

Even if a particular interaction or a pathway is identified, the transient nature and moderate affinity of many DMIs often leads to a lack of structural information, which in turn makes it difficult design molecules that mimic the natural interfaces [25, 26, 37–43]. In many cases the protein domain of the target motif may also be naturally disordered, or unfolded unless the conformation is stabilized through binding [5].

DMI interfaces are modular, and present in a wide number of proteins, cellular localizations and contexts. This means that proteins involved in protein-protein interactions can often be ‘promiscuous’ binders – targeting several proteins using the same motif [2, 44]. They also may be common to other proteins that display high sequence identity [2, 26, 42, 45, 46]. Finding inhibitors with an acceptable level of specificity is therefore difficult to achieve, and often good candidates show unforeseen toxicity by inhibiting multiples pathways [47].

### Structural and physiochemical properties of the interfaces make them difficult to target with classical screening methodologies

Typical protein-protein interaction interfaces tend to be large, flat and mainly hydrophobic, where punctual electrostatic interactions are key for the binding [1, 3, 6, 28]. Only a few amino acids in these interfaces are critical to the binding and recognition. These residues, often referred to as hotspots, are major determinants of affinity and specificity, but at the same time allow flexibility to fit particular modifications [2, 3, 25, 26, 42, 43].

**Fig. 2:**
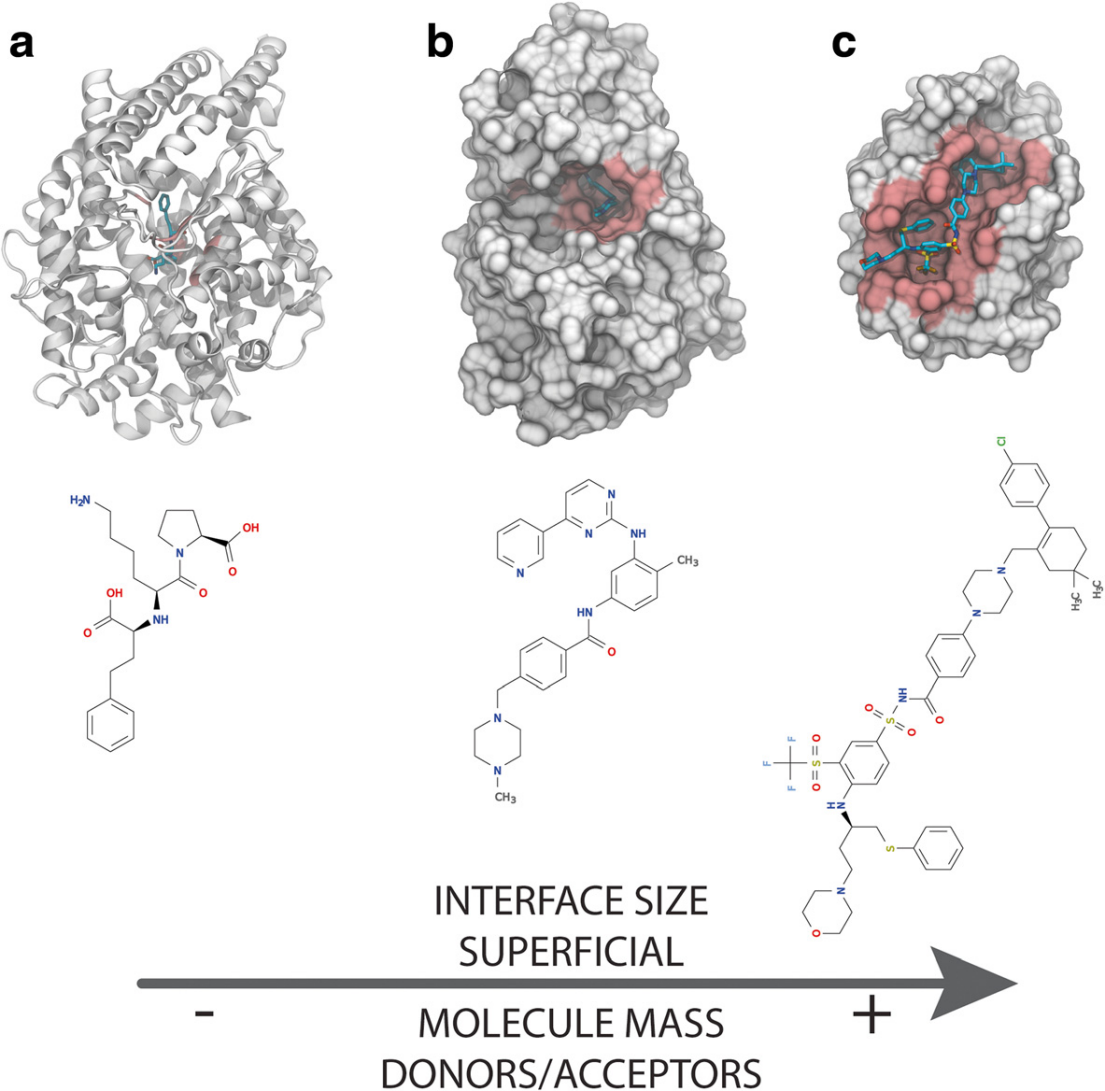
Structural comparison between a drug within Lipinski’s rules (Lisinopril), a kinase inhibitor (Imatinib) and finally a protein-protein interaction inhibitor (ABT-263). Panel **a** (PDB: 1O86); Crystal structure of the drug Lisinopril in complex with angiotensin-converting enzyme. Lisinopril inhibits angiotensin-converting enzyme. This drug is used to treat hypertension and symptomatic congestive heart failure, and to prevent progression of renal disease in hypertensive patients with diabetes mellitus and microalbuminuria or overt nephropathy. Angiotensin-converting enzyme is represented in cartoon representation colored in grey with the active site in red. The drug is shown in licorice representation. Panel **b** (PDB: 2HYY); Crystal structure of the Human Abl (Abelson murine leukemia viral oncogene homolog 1) kinase domain in complex with the inhibitory drug Imatinib (licorice representation). Imatinib, Gleevec (USA), or Glivec (Europe/Australia) is a kinase inhibitor used to treat chronic myelogenous leukemia (CML), gastrointestinal stromal tumours (GISTs) among other malignancies. Abl kinase domain protein surface is colored in grey with the active site in red. Imatinib is represented in licorice representation. Panel **c** (PDB: 4LVT); High-resolution crystal structure of the drug ABT-263 (licorice representation) bound to Bcl-2 (grey surface with interface highlighted in red). ABT-263 or Navitoclax is an orally bioavailable small molecule inhibitor of Bcl-2 family proteins currently in clinical trials for the treatment of lymphomas and other types of cancer. Bcl-2 is shown as a grey surface, where the motif recognition interface is highlighted in red. ABT-263 is represented in licorice in the complex. A 2D representation of each drug is displayed in the lower section of the figure

In general these geometric and physiochemical properties are incompatible with the classic small molecules that satisfy Lipinski’s rule of five, with good pharmacokinetics properties. This is shown empirically given the low ratio of success by high-throughput screening in identifying compounds [24, 25, 27, 29]. The traditional HTS compound libraries contain scaffolds without appropriate physicochemical properties to maximize binding complementary with the PPI interfaces [6, 23, 37, 39]. It is also the case that in order to target these large and complex interfaces with enough specificity, we need to design larger compounds (Fig. 2). Increasing size involves new challenges, for instance the rise of the entropic penalty to bind (less potential to reach lower affinities) [33, 48] as well as poor cell delivery [3, 28, 30–32].

### Reaching the interactions is not easy. Intracellular targets

A common scenario is that a lead shows promising binding affinity, but is not active in cell-based or in vivo assays. One example is the inhibitor for the transcription factor HIF-1 PAS domain [23]. In order to target intracellular interactions, the inhibitor must be capable of both surviving in an environment exposed to proteases, immune response, etc., and crossing the cell membrane. As mentioned, DMI interfaces tend to be relatively large, and thus their inhibitors are often large as well (more complex molecules, even peptides or peptidomimetics). These molecules have more difficulties in passively crossing the membrane or surviving in the cell. In addition, DMI are highly localized inside the cell, adding an extra challenge for the molecule to hit its target with enough local concentration to trigger a therapeutic response.

### Main methodologies to inhibit PPIs: targeting protein-protein interactions with small molecules

Classic drug development works with small, chemically manufactured active molecules. These molecules have a wide range of desirable properties for drug discovery. For instance, they are relative easy to synthetize and manipulate, and in general they have a good cellular uptake. However, these molecules tend to bind better with smaller and deeper grooves than the DMI interfaces [2]. Therefore targeting DMI with small molecules required an evolution in classical methodologies to fit these new challenges [29].

This has been accomplished by increasing the complexity of drug molecules, in order to fit the properties and sizes of the DMI interfaces. At the same time, growth and refinement of the lead was carried out with a complete structural description of the natural binding motif. Precise identification of the motif, and of hot spots involved in the interaction, were critical to maximize specificity and affinity while keeping the compound size reasonable.

### Targeting DMI with HTS. Example MDM2/p53

In many cases there is little information available on the targets, and High Throughput Screening (HTS) is a more effective approach. However, as we mentioned, HTS has shown limited success against PPIs due to a bias of scaffolds in the compound libraries [6, 23]. Notwithstanding, a specific inhibitor for the MDM2/p53 interaction was discovered. Success was partially due to the fact that successfully inhibited interactions are domain-motif interactions, and the resulting molecule was mimicking the peptide motif. Thus through a considerable efforts in in medical chemistry and careful structural considerations, a high affinity binder was generated [30–32].

The tumor-supressor gene p53 induces cellular apoptosis in response to DNA damage, avoiding possible tumorigenesis. Although many human cancers have a mutation or deletion in p53, in a significant proportaion of cancers the function of p53 is inactivated by a deregulated expression of the onco-protein, HDM2 (an E3-ubituitin ligase also know as MDM2), promoting tumorigenesis and poor response to cancer therapy [20]. Therefore, the interaction MDM2/p53 has been a potential target for chemotherapeutics agents. MDM2 binds to a mostly hydrophobic 15 reside α-helix region at the C-terminus of p53. Alanine scanning of the 15 residues in p53 identified 3 residues with a major contribution to the binding; PHE19, TRP23 and LEU26 [49]. The crystal structure of MDM2 bound to the p53 helix reveals how these residues, in the centre of the interface, fit in a small pocket in MDM2. The existence of such a pocket on MDM2 raised the expectation that small compounds would block the interaction. A subsequent HTS and a medicinal-chemistry effort at F.Hoffman-LaRoche led to the discovery of several inhibitors. The most promising one was Nutlin, which mimics interactions of the p53 peptide in the pocket of MDM2. Despite early promise, Nutlin was ultimately unsuccessful in clinical trials [50]. However, the crystal structure of this small compound, together with a detailed description of the binding motif, facilitated the development of new inhibitors. Recently, using this information as template, in a combination of rational design, computational modelling, structural screening and biophysical techniques, several new classes of inhibitors were developed. These included spiroxindole-base molecules (MI-219 and its posterior improved version MI-888) [51], morpholinones (AM-8553) [52], piperidiones (AMG-232) [53] and sulphoanomide (NSC279287) [54]. All of them had sub-nanomolar affinity to MDM2, good pharmakokinetic properties, tumour suppression and are currently in different phases of clinical trial [55].

### Fragment-based methodologies. Example Bcl2/BH3

A successful alternative to HTS are fragment-based drug discovery strategies. These methodologies are based on identifying small chemicals, which may bind weakly at different spots on the target interface, and then combining them to produce a single lead with higher affinity and specificity. These approaches allow the construction of larger, more complex compounds, more likely to block specifically DMI interfaces. For instance, using Fragment-based drug discovery a potent inhibitor for B-cell lymphoma 2 (Bcl-2) has been discovered.

The Bcl-2 family proteins are important regulators of the cellular apoptosis mechanism. Aberrations in this decision mechanism can enable cancer cells to evade death [56]. For instance, overexpression of the antiapoptotic Bcl-2 genes is frequently observed in solid human tumours. Inhibition of relevant members of this family therefore represents a novel and promising strategy for new types of anticancer drugs. A key element in the signalling process of Bcl-2 family members is the direct binding of a protein containing a BH3 domain (Bcl-2 homology domain 3) [18].

Their interaction mode consists of a slight groove on the multidomain protein, serving as a receptor site for docking of the signature α-helical BH3 domain. For example, Bcl-2 and Bcl-X_L_ inhibit apoptosis by binding a 16 residues α-helical portion of the pro-apoptotic protein Bcl-2 antagonist/Killer (BAK) or a 26 residue α -helix portion of Bcl-2 antagonist of Cell Death (BAD). This structural information was completed by identification of the hot-spots at the interface through alanine mutational scanning [57] Much effort was then focussed on the development of synthetic inhibitors of these protein-protein interactions using small molecules that mimic the interactions of the α-helices of BAK and BAD. Classical approaches, such as high-throughput screening of a historical compounds, failed to provide high-affinity compounds [29], and several other approaches have been developed with only partial success [29, 58]. Finally, a dual inhibitor of Bcl-2 and Bcl-X_L_ was discovered by Rosenberg, Fesik and co-workers [4, 59–62]. The successful strategy was to apply what has since become known as fragment-based drug discovery [38, 41, 63, 64]. The methodology consisted of identifying two or more simple molecules that bind adjacent, but without overlapping at the interface, and use the structural information provided by these fragments as a guide to build one unique compound. Rosenberg and Fesik used nuclear magnetic resonance spectroscopy for both screening and connectivity-guiding aspects in discovery of the high-affinity organic compound, ABT-737 (obatoclax). This small molecule binds to the BH3 domains with high affinity and inhibits interaction with the pro-apoptotic proteins BAX and BAK. It was also active in cell-based assays and in tumour xenograft models in animals. ABT-263 (navitoclax), a derivative of the former molecule is currently in clinical trails (Fig. 2c). Recently, ABT-199 was developed by Sours and coworkers [65]. This structure based-redesigned version of ABT-263 has shown suppression of tumor growth and a higher specificity for Bcl-2 without losing affinity [66]. However, the enormous tumour lysis after treatment with ABT-199 caused serious complications in patients, leading to suspension of the clinical trials and reconsidered doses and route of administration [67]. Recently, new clinical trials reported promising results and ABT-199 is close to FDA approval. This will be a real step forward in chronic lymphocytic leukemia (CLL), and potentially several other forms of leukemia, lymphoma, and myeloma.

### Main methodologies to inhibit PPIs: targeting protein-protein interactions with biologics

In the previous section we showed how natural protein interactions can be used as a template to design synthetic molecules that imitate the natural interactions. It is also true that peptides and proteins are themselves a viable alternative to small compounds for targeting PPI motifs, because of their high selectivity, low toxicity and predictable metabolism [2, 3, 30–32, 34, 44].

Despite these features and the number of available advanced methodologies for their synthesis and study, peptides have many intrinsic limitations for use as drug molecules. Limitations include lack of proteolysis stability, relatively low affinity, poor cell-penetrability and short plasma half-life [24, 25, 30, 34, 68, 69]. Fortunately, there are many methodologies to address these issues and provide promising drug candidates.

### Peptidomimetics. Example IAPs

One promising approach is the design of peptidomimetics molecules. These molecules typically derive from existing peptides and tend to conserve a protein-like chain, but with its chemical structure modified in order to adjust the molecular properties to become more drug-like. These modifications involve the introduction of non-canonical amino acids [70–72], chemical stapling α-helix conformations [45, 50, 73–75], modifying the chirality [76–79] and cyclization [80–83].

The Inhibitors of apoptosis (IAPs) proteins are a family of negative regulators of apoptosis. IAPs, first identified in baculoviral genomes, bind to caspases – enzymes response of cellular death, through physical interactions mediated by the baculovirus IAP repeat domain (BIR) [21, 31, 32]. These domains recognize and inhibit caspase activity, stopping cell death. The most characterized member is X-linked inhibitor of apoptosis protein (XIAP), which appears to be frequently deregulated in cancer. Thus, inhibition of the BIR domain-caspase interaction becomes a promising approach towards treating cancer.

XIAP contains three consecutive BIR domains at the N-terminus, but only two are involved in caspases inhibition. BIR2 binds and inhibits Caspase-3 and Caspase-7, and BIR3 is involved in Caspase-9 inhibition. Nevertheless, repression of XIAP activity can be achieved by the endogenous mitochondrial protein; second mitochondria-derived activator of caspases (Smac/DIABLO). Smac/DIABLO bind at BIRC3 domain of XIAP releasing capases and re-activating apoptosis using a conserved tetrapeptid motif (AVPI) [32, 68, 84–86]. The isolated 4-mer peptide derived from Smac also binds to XIAP with 3 digit nanomolar affinity, and the crystal structure revealed the tetrapeptide binds to a surface groove present in the BIR domain [68, 73]. Following the discovery and characterization of the Smac sequence, several groups used the information to develop new peptides capable of binding to XIAP with refined affinity [73, 87, 88]. The importance of each position was also established from peptide libraries. However, the early short peptides, though displaying relatively high affinity, lacked favourable physiochemical properties, and efforts to find a lead by HTS were ineffective [73].

**Fig. 3:**
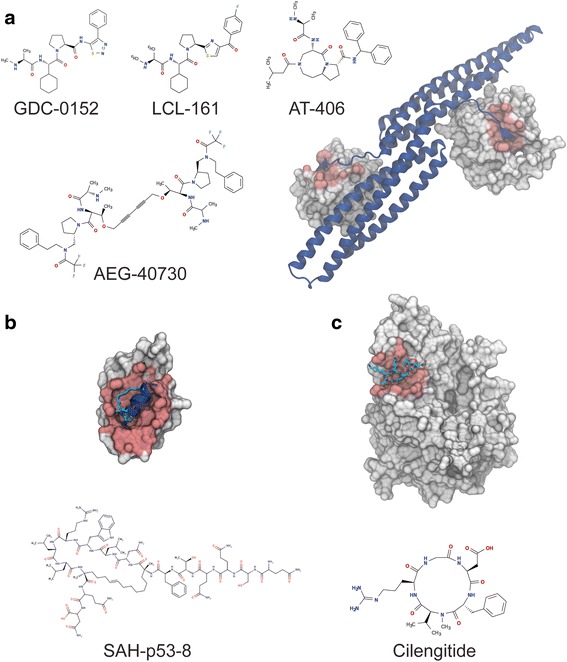
Targeting Protein-Protein interactions with Biologics. Panel **a**; Crystal structure of the complex of Smac homodimer protein with two XIAP BIR3 proteins (PDB: 1G73). The XIAP proteins are shown as a grey surface, with the motif recognition interface highlighted in red. The dimeric Smac is represented in blue cartoon representation. Next to the complex, the 2D molecular representation of the peptidomimetics of Smac in clinical trials is shown. Panel **b**. Structure of the Stapled p53 Peptide (SAH-p53-8) Bound to Mdm2. (PDB: 3V3B). MDM2 protein surface is displayed in grey with the motif recognition interface highlighted in red. The Stapled peptide is shown as a cartoon representation in blue and the covalent linkage is displayed in licorice representation. Panel **c**. Structure of the αvβ (3) integrin bound to the Arg-Asp-Gly (RGD) motif of fibrinogen. (PDB: 2VDR). The integrin surface is colored in grey, and the recognition motif interface is highlighted in red. The Fibrinogen binding motif is represented in licorice. Below the complex structure, a 2D representation of the protein-protein interaction macrocyclic inhibitor Cilengitide is shown

A successful approach was to develop the tetra-peptide into peptidomimetic molecules. A systematic examination of peptide tolerance to substitution by each amino acid for non-canonical amino acids led to different compounds with more drug-like properties [84–86]. Shortly after the first reports appeared detailing Smac-derived peptidomimetic, a set of patents emerged that disclosed dimeric derivatives of these peptidomimetics. The dimeric Smac peptidomimetics are capable of interacting simultaneously with BIR2 and BIR3 domains of XIAP to induce a more potent response than the monovalent [73]. Currently, four compounds (AEG-40826/HGS-1019 Aegera therapeutics; AT-406, Debiopharm and Ascenta Therapeutics; LCL-161, Novarits; GDC-0152, Genentech) are in different phases of clinical trial [29, 73, 87, 88] (Fig. 3a).

### Stapled peptides example MDM2/MDMX

There are several good inhibitors of the complex MDM2-p53, leading to restoration of p53 activity. However, these molecules are only active against MDM2, and some authors have argued that dual inhibitors of MDM2 and MDMX are needed to expand the range of tumours that can be treated. [89]. MDMX, also known as MDM4, shares a high degree of sequence similarity with MDM2 and it is another negative regulator of p53 activity [90]. Despite its homology with MDM2, the mechanism of MDMX is not well understood [91, 92]. Nutlin and other small molecules are incapable of disrupting MDMX-p53 complexes; the appearance of chemoresistance appears to be a result of MDMX overexpression [8, 19, 93].

While the evidence suggests that p53 binds to MDMX through the same interface, designing a small molecule able to target both proteins proved to be a challenging goal. The most successful strategy was to convert the C-terminal p53 α-helix from the native p53– MDM2/X complexes to a more stable molecule by peptide stapling [89, 94].

The term “staple” reflects the covalent linkage of two non-consecutive α − methyl- amino acids through its all-hydrocarbon tethers. This methodology was initially developed Gubbs and coworkers to create macrocyclic peptides [95] and refined by Verdine and coworkers with the intention of stabilizing helical peptides [75]. The α-helix represent a common structural motif in protein-protein interactions, but a synthetic helical peptide can lose this secondary structure, decreasing the affinity through entropic affects. The stapled helices have been proven to maintain their structure and biological activity, and at the same time increase cellular uptake and protease resistances, all of them favourable drug-like properties [96].

Bernal and coworkers applied this strategy of chemical stabilization to the α-helix peptide of p53, where they designed and studied 16 different variants [97, 98]. The variant SAH-p53-8 was demonstrated to have dual activity against MDMX and MDM2. Later, after some iteration over SAH-p53-8, Sawyer and coworkers reported an enhanced version with drug-like properties [99]. This new stapled-peptide has shown antitumor activity where MDM2 and MDMX were overexpressed, both in cell assays and in vivo. In addition, it exhibited enhanced cell penetration and in vivo half-life. In 2013, Aileron Therapeutics completed the first phase of clinical trial for a stapled peptide and it is currently in a further stage of trial [96] (Fig. 3b).

As mentioned above, helices are a popular structural motif in protein-protein interactions and therefore the potential of this approach to discover new inhibitors is really promising. Not only can peptide affinity be improved, but also its pharmokinetic properties. Therefore numerous studies have proposed stapled peptides as lead molecules, including BH3, Ras-Sos and other oncogenic targets [100–103].

### Macrocycles. Example cilengitide

Linear peptides in solution can explore an enormous number of conformations. This entropic behaviour is often related with poor selectivity and affinity because there is a large entropic penalty to adopt the bioactive conformation [104]. One strategy to reduce the conformational space is cyclization of the peptide, analogous to stapling above. A collateral effect of cyclization is that cyclic peptides show higher resistance to proteases [58]. Development of the Cilengitide is an example of a successful application of this approach. In addition to cyclization, other modification were made that introduced conformational restrictions, to increase affinity and specifity of the pentacyclic peptide to targeting αvβ3 and αvβ5 integrin Receptor. This example is remarkable since the structure-activity development of this lead was carried out mainly without any structural information of the complex [82].

Integrins are heterodimer receptors that are crucial in cell-adhesion, providing signalling into the cell in case of proper adhesion. Among other processes they play a key role in the angiogenesis and metastasis of solid tumours being a promising target for cancer therapy [22]. A subgroup of the integrins recognise and bind proteins in the extracellular matrix through the tripeptide motif, Arginine, Glicine and Glutamic acid (RGD) [82]. While flanking amino acids appear unimportant for binding, Integrins can discriminate between different targets, suggesting a secondary mechanism of recognition. It was later shown that integrin receptors recognize a distinct conformation of the RGD motif, modulated by the target protein [82]. Preliminary experiments with disulphide cyclized peptides showed how the cyclic peptides inhibit specifically only vitronectin mediated adhesion and do not affect fibronectin adhesion, while the linear peptide indiscriminately inhibited both processes [105]. Nevertheless, these experiments validated the conformation-dependent recognition mechanism, despite a lack of structural information on binding.

In order to investigate which conformations were preferred by the Integrins, Kesseler and collaborators, explored the conformation space of pentapeptides (RGDFV), and hexapeptides (RGDFVA) containing the binding motif. They controlled the conformational space of the library by generation of peptides where one amino acid was a systematically substituted by its D-form. This substitution, promotes a conformational change without changing the chemical nature of the sequences. This approach, later named “spatial screening”, led to the discovery of a specific inhibitor for αvβ3 integrin Receptor, a promising starting point to discovery a new drug [106]. This cyclic-pentapeptide was used as a framework for a wide range of different substitutions, and finally introduction of N-methyl amino acids in the sequence led to the discovery of Cilengitide (Fig. 3c). Unfortunately, recent results from phase III clinical trials showed a non-significant increase in patient survival in patients diagnosed with glioblastoma and methylated MGMT (O^6^-methylguanine–DNA methyltransferase) gene promoter. Currently Cilengitide has entered in phase II trials with glioblastoma patients with unmethyleted MGMT gene promoter [107].

### Outlook and new trends

#### Screening

Perhaps the most important lesson learned from successful PPI inhibitors is the value of quality structural information describing the interaction, and accurate knowledge of the binding motif. When little information about the targets is available however, HTS is the better approach. As already mentioned, HTS has shown limited success against PPIs due to a bias of scaffolds in the compound libraries [6, 23]. For this reason, current libraries are focussed on maximizing the molecular complexity and diversity rather than complying with the rule of five [25, 26, 38, 41, 42]. These new libraries of natural and synthetic compounds have demonstrably been a more efficient approach for the discovery of small molecules capable of interference with PPI motifs [3, 43, 60–62, 108, 109]. Recently, a library of 10,000 compounds was screened for potential inhibitors of Min1-PDZ (involved in the synaptic function and target to treat pain) identifying several lead molecules [110]. Lately, several companies, e.g. ASINEX, OTAVA Chemicals, made commercially available libraries specifically designed to target DMI. Moreover, there are successful studies using virtual compound libraries specifically designed to target a family of domains, as shown by Optiz et al. targeting proline rich binding domains [111, 112].

In parallel, screening methodologies are evolving as well to achieve better ratios of success targeting PPI motifs. As we explained in a former section, fragment based screening has shown as a successful approach targeting DMI. However, This methodology requires high fragment concentrations for a detectable occupancy, increasing the possibility of unspecific interactions and false positives [113]. This limitation can be overcome with a variant of fragment based screening known as Tethering. This methodology, first reported by Erlanson and coworkers [114], relies on the amplification of fragment affinity, by reversible covalent bond formation between fragment and target. Tethering methodology requires both, a library of fragments with a disulfide group, and a cysteine residue next to the interface. The screen is then performed under moderately reducing conditions to promote thiol-disulfide exchange with the target. A fragment with favourable interactions with at interface will then stay at the interface longer than other fragments, shifting the equilibrium and becoming the most abundant species. Mass spectrometry analysis can subsequently reveal which fragment has the highest protein affinity [115]. For instance, Braisted and co-workers employed the tethering approach to identify small molecules capable of binding to IL-2 (interleukine-2), and modulating the activity of its hetero-trimeric receptor. They prepared and validated 11 different cysteine mutants to cover the entire interface of this DDI, and screened a library of 7000 fragments for each of them. By assembling all of the information provided by tethering screening, SP4206, a compound with nanomolar affinity, was finally synthetized [115]. Furthermore, tethering has been shown to be a valid approach for targeting both DDI and DMI interactions. Wang et al. have reported the application of tethering to discover small molecule ligands for the KIX domain of the master co-activator CBP/p300.9 [116]. Nonetheless, continuous improvement is an on-going effort to improve this methodology. Recently, Lodge et al. have shown how tethering can be performed rapidly and inexpensively using a homogenous fluorescence polarization (FP) assay that detects displacement of a peptide ligand from the protein target as an indirect readout of disulphide formation [117].

Another approach is to screen directly using cyclic peptide libraries genetically encoded in cells [81, 118]. The classic two-hybrid system can be altered to link cell growth to the disruption of a complex rather than the complex formation, a method called reverse two-hybrid system (RTHS). In parallel, cells are transformed with an extra vector that encodes for a peptide of a combinatorial library and the necessary proteins to perform the intracellular synthesis of cyclic peptides (SICLOPPS) [81, 118]. This methodology allows the discovery of cyclic peptide-base dissociative inhibitors through the combination of SICLOPPS technology with RTHS. It has been applied to different proof of concepts resulting in cyclic peptides with comparable affinity to known inhibitors, and others with unprecedented binding modes [81, 118].

Peptides and peptidomimetics – and even proteins – present a completely new set of challenges to solve, but there are proposed solutions with promising preliminary results. Probably the largest challenge for employing biologics as inhibitors of intracellular interactions is cellular uptake.

### Delivery and pharmacokinetic properties

Recent discovery of potent therapeutic molecules, which did not reach the clinic due to poor delivery and low bioavailability, has made the delivery of such molecules a key issue in therapeutic development. A wide range of different strategies are being explored to achieve this, as such, lipid-derived compounds (pepducins and liposiomes) [119, 120], polymeric nanoparticules [121], inorganic carriers [122, 123], super charged proteins [124], deactivated pathogen toxins [125, 126] and, most commonly, cell penetrating peptides (CPP) – like the transactivatior of transcription (TAT) of HIV-1 [127, 128]. CPP mechanisms are still poorly understood and the subject of strong controversy [127, 129]. Other strategies that have proven successful are peptides with reversed chirality and stapled peptides. These approaches not only improve cell permeability, but also reduce proteolysis and enhance metabolic stability [27, 29, 59, 130]. Finally, another limitation arises from the poor pharmacokinetic properties of these types of molecules. Peptides present low toxicity and predictable metabolic properties, but are easily degraded either in cells or blood. The addition of non canonical aminoacids, D-forms, and punctual modifications as such as N-Methylation of peptide bonds to the candidates, have proven to be powerful approaches in increasing peptide drug potential [131–133]. However, target identification still presents a major bottleneck in the discovery of new inhibitors [58]. Screening methods to discover new targets modulated by DMIs.

### Identification of new targets

The initial research of a drug, often occurring in academia, generates data to develop a hypothesis that the inhibition or activation of a protein or pathway will result in a therapeutic effect in a disease state. The outcome of this activity is the selection of a target, which may require further validation prior to progression into the lead discovery phase in order to justify a drug discovery effort.

The complexity of PPI networks make it difficult however, to identify clear targets, even using high-throughput methods such as yeast two-hybrid (Y2H) or affinity-purification mass spectrometry (AP/MS). While other methodologies, like peptide arrays, split-protein systems [134, 135], and peptide-phage display [136] can identify DMI, they too have their limitations. Peptide arrays have very limited coverage, because the number of peptides that can be printed on an array and conventional phage libraries display can identify biophysically optimal ligands of modular domains, but this approach can exhibit a hydrophobic bias and may not be ideal for detecting natural binders [137]. Thus, there is a need for alternative approaches for the identification of relevant domain–motif interactions.

Ivarsson and coworkers use custom oligonucleotide arrays to construct defined phage display libraries comprising the entire human and viral C-terminuses found in Swissprot. Oligonucleotides encoding the c-terminal heptapeptide sequences were printed on microarray slides, PCR amplified, and cloned into a phagemid designed for the display of peptides fused to the C-terminus of the M13 major coat protein p8. The libraries were used in binding selection with PDZ domains and the selected pools were analysing by next-generation sequencing on the illuminia platform. This approach allowed them to screen several orders of magnitude larger than peptide arrays, avoid the bias inherent in random exploration, and scan natural interactions. Using this approach they identified known and novel human and viral ligands, and validated candidates in vivo and in vitro [40].

## Conclusions

Discovery and subsequent refinement of PPI inhibitors with strong affinity has proven to be a challenging, though not impossible, quest. A number of inhibitors were discovered by close examination of the interactions and precise identification of DMI hot-spots. Likewise, the adaptation of techniques used to investigate specific characteristics of PPIs has been critical to the successful identification of new inhibitors.

Several inhibitors for DMI are currently in the late stages of clinical trial and more are expected to follow. Furthermore, inhibitors that failed during late stages of clinical trials, such as Nutlin and Cilengitide, have a second chance to be used in combination therapies [138–140].

New approaches and new targets are currently emerging, and new developing technologies of the post-genomic era may yield more advanced methodologies for PPI inhibition. In the coming decades we may plausibly reach the capability to disrupt PPI networks and modulate signalling pathways *at libitum*, and develop therapeutic solutions to individual pathologies.

## Abbreviations

Bcl2: B-cell lymphoma 2
BIR: bacuolovirus inhibitor of apoptosis repeat
DDI: domain-domain interactions
DMI: domain-motif interaction
HDM2: human protein double minute 2
HTS: high throughput screenings
IAPs: inhibitors of apoptosis
IL-2: interleukin 2
MDM2: murine double minute 2
PPI: protein-protein interactions
RTHS: reverse two-hybrid system
SICCLOPPS: split-intein circular ligation of peptides and proteins
XIAP: X-linked inhibitor of apoptosis protein
Y2H: yeast two hybrid
